# Tunga Penetrans: Painful Lesions on the Feet—The First Imported Case from Guinea-Bissau

**DOI:** 10.1155/2010/681302

**Published:** 2010-12-16

**Authors:** A. Rosmaninho, S. Vilaça, V. Costa, A. Sarmento, I. Amorim, M. Selores

**Affiliations:** ^1^Serviço de Dermatologia, Centro Hospitalar do Porto, HSA, Edifício das Consultas Externas, Ex CICAP, Rua D. Manuel II, 4099-001 Oporto, Portugal; ^2^Serviço de Infecciologia, Hospital de São João, 4200-319 Oporto, Portugal

## Abstract

Tungiasis is an endemic disease in certain poor areas around the world. Imported infestations in travelers are becoming more frequent and can lead to considerable morbidity. We report a case of a 50 year-old-man who returned from a trip to Guinea-Bissau with an infection caused by *Tunga penetrans*.

## 1. Introduction

Tungiasis is a cutaneous parasitic infection caused by the penetration of a sand flea into the skin of its host. In Europe the disease is almost exclusively seen in travelers returning from endemic areas. With the increase of foreign travel and immigration the chances of physicians encountering this tropical disease is rising, and an early diagnosis is required since several complications can be seen if the disease is neglected.

## 2. Case Report

**Figure 1:**
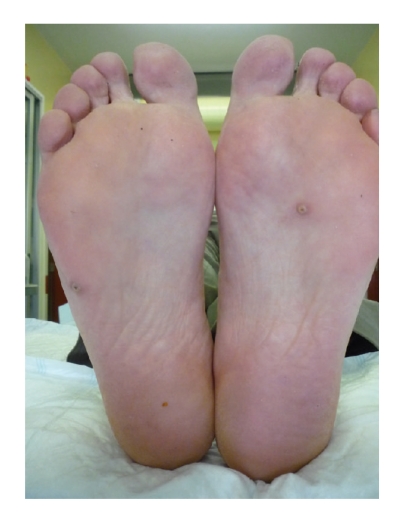
Papular lesions with a small brown-black central core were distributed on the soles and lateral aspect of the toes.

**Figure 2:**
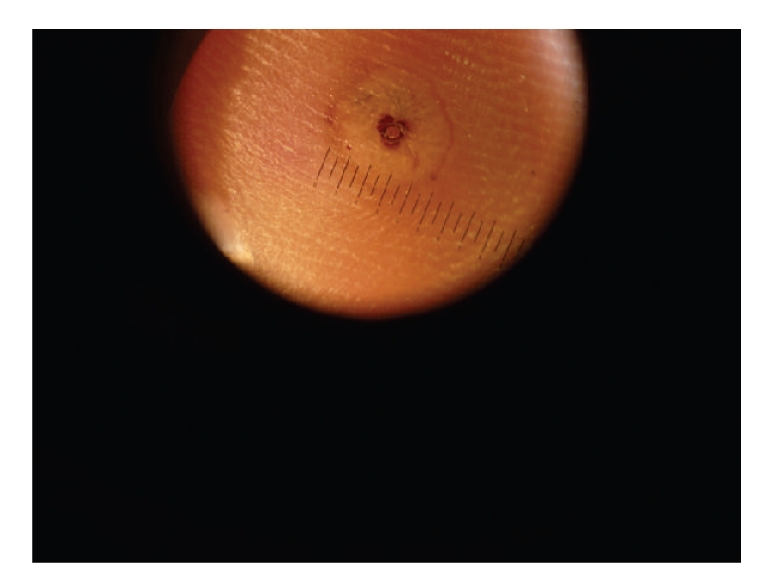
Dermoscopy findings in our patient. A black area with a plugged opening in the center surrounded by a whitish halo and by a peripheral slight reddening (surrounding inflammatory zone).

**Figure 3:**
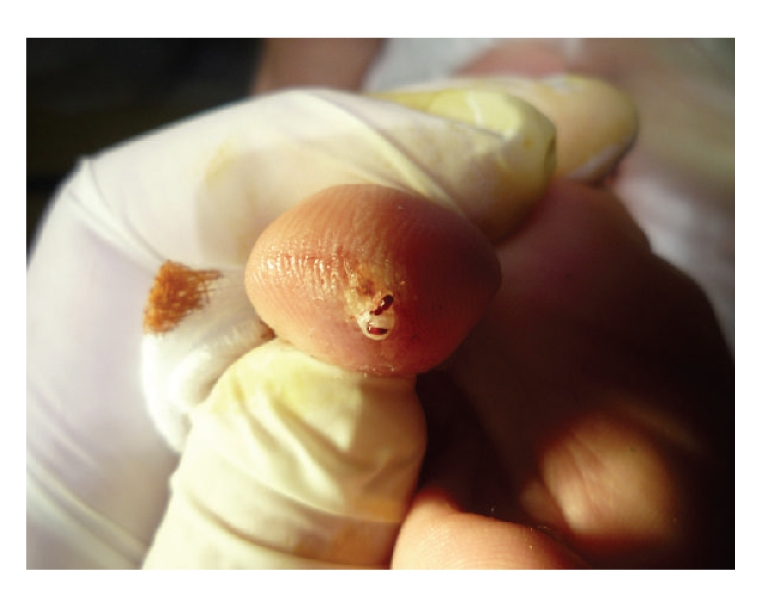
Excision of the lesions.

**Figure 4:**
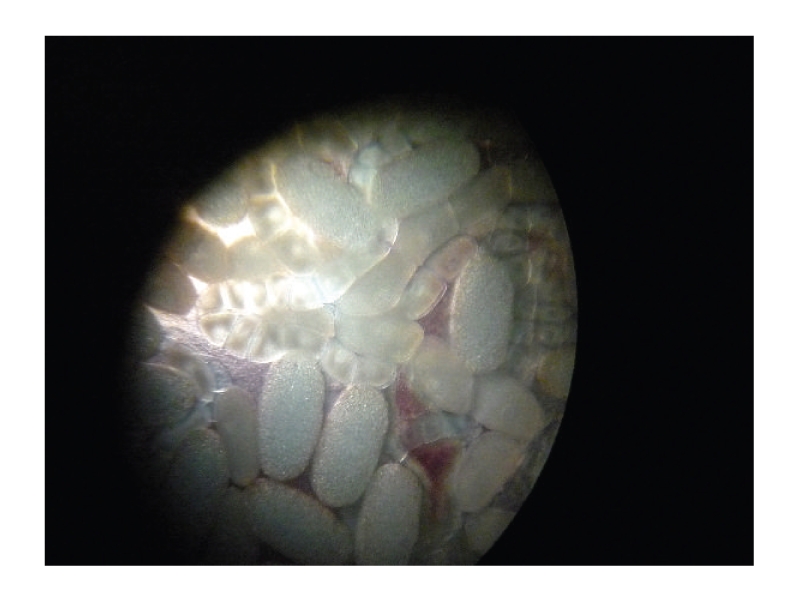
Optic microscopic view of *Tunga penetrans* eggs after excision of the flea from the foot lesion.

A 50-year-old male presented with a fifteen day history of multiple painful lesions on the soles. He reported a “burning sensation,” pruritus, and progressive pain with marked limitation in walking. He denied history of insect bite or trauma to the affected area. He had been traveling in Guinea-Bissau, were he walked in bare feet on a beach. There were pigs and goats. His prior medical history was unremarkable. On physical examination multiple 1 cm, round, white papular lesions with a small brown-black central core where distributed on the soles and lateral aspect of the third and fifth toes of the right and left foot, respectively (Figures 1 and 2). A diagnosis of tungiasis was suspected based on the clinical history and physical findings. Surgical incision of the lesions with deep removal of the content was performed under local anesthesia (Figure 3). The content from the wound was analyzed throught optic microscopy and showed multiple *Tunga penetrans* eggs (Figure 4). The patient went on topical fusidic acid cream with healing of the lesions.

## 3. Discussion

Tungiasis is an ectoparasitosis caused by the penetration in the skin of the gravid female of the sand flea *Tunga penetrans*. Other popular designations for the parasite includes: *chigga*, *chica*, *jiggers*, *bicho do pé*, *moukardam,* and *pico*. The flea infestation is associated with poverty and is endemic in some Caribbean, South America, Asia, and Africa countries [1]. The current epidemiological situation on the African continent is not well known and is mainly based on anedoctal observations. Recent studies in Nigeria, Cameroon, and Brazil reported similar high prevalence of tungiasis (45%, 49%, and 51%, resp.) [2–4]. In the study of Collins G. et al, the prevalence of the disease in a rural Cameroon area was greater in children than in adults. This was seen mainly because of the use of open or damage shoes. In addition, children work on the farm from a young age adding a greater exposure to infection [5]. Human infections are linked to important sociocultural factors associated with poverty: the practice of walking barefoot or wearing only sandals, sandy floor inside the house, living in a house made of palm products, lack of personal and soil hygiene, and the free movement of animals (pigs, dogs, and rats) between and into houses. Tunga infection affects many species of domestic animals, in addition to humans. The pig has been considered to be the main reservoir, but *Tunga penetrans* has been reported in cows, dogs, cats, goats and rats. In Europe the disease is rare with only one autochthonous case being reported [6]. Most of the European reports belonged to travelers returning from endemic areas. As far as we are aware, this is the first case of *Tunga penetrans* infection imported from Guinea-Bissau. The transmission of the flea occurs by walking barefoot on the sandy soil of disease-endemic regions. In the skin the flea burrows a cavity with the head turned toward the upper dermis, in order to feed on the host's blood and begins to produce eggs (150–200 eggs over a period of 2-3 weeks) [7]. Only the end portion of the abdomen extrudes and the flea can reach a diameter of 1 cm. The penetration is usually asymptomatic with pain developing only when the flea increases in size. The clinical findings depend on the stage of infestation (Fortaleza Classification). From penetration of the parasite into the skin to the healing it takes 4–6 weeks [8]. Infestations, usually present with papular or nodular lesions, either single or multiple, white or gray in color with a small brown central opening, corresponding to the posterior portions of the abdomen. Plantar wart-like lesions, as well as pustular, ulcerative bullous lesions have been described [9–11]. Most lesions are localized on the feet and toes, mainly in the subungual and periungual areas, but other ectopic localizations such as hands, back, buttocks, wrists, perineum, and breasts have been documented [12, 13]. Several dermoscopic findings have been reported as useful tools for an early diagnosis. It includes a black area with a plugged opening in the center (corresponds to the opening of the exoskeleton), a peripheral pigmented ring (corresponds to the posterior part of the abdomen), gray-blue blotches (corresponds to the eggs in the abdomen) [14], and a radial crown (a zone of columnar hemorrhagic parakeratosis in a radial arrangement) [15]. Diagnosis of tungiasis is based on the characteristic aspect of the lesions in a patient who recently visited an area where the disease is endemic and can be supported by the dermoscopy findings mentioned above. An early diagnosis reduces the possibility of bacterial infection complicated by ulceration, cellulitis, lymphangitis, tetanus, osteomyelitis, gangrene, and spontaneous amputation of the toes [16]. Surgical extraction of the flea under sterile conditions and a topical antibiotic applied afterwards are considered the treatment of choice. During the excision care should be taken to prevent tearing of the flea and to avoid parts of the flea being left behind due to the risk of severe inflammation ensuing. Topical treatment includes: cryotherapy, electrodesiccation, formaldehyde, or chloroform. A randomized trial showed that topical metrifonate, ivermectin, or thiabendazole can each reduce the number of lesions [17]. Oral ivermectin has been reported to be effective, although recently Heukelbach et al reported that its efficacy was practically nil [18]. Systemic treatment with oral thiabendazole has also been reported to be successfully used in patients with generalized infestations [19]. Prevention of the infestation is essential and includes the use of closed footwear, the use of repellents and immediate extraction of embedded fleas. In endemic areas replacing sand and mud floors with concrete or tiled floors as well as avoiding the contact with animals that could be infected are important measures to prevent the spread of infestation [3, 20]. In conclusion, tungiasis is a rare disease in nonendemic areas and with the global warming, increased foreign travel and immigration from endemic areas the parasite may further disseminate to new geographical areas such as Europe. Thus, physicians should be familiarized with this parasitic disease, since an early diagnosis and an adequate treatment can prevent serious complications.

## References

[1] Heukelbach J, Sales De Oliveira FA, Hesse G, Feldmeier H (2001). Tungiasis: a neglected health problem of poor communities. *Tropical Medicine and International Health*.

[2] Ugbomoiko US, Ofoezie IE, Heukelbach J (2007). Tungiasis: high prevalence, parasite load, and morbidity in a rural community in Lagos State, Nigeria. *International Journal of Dermatology*.

[3] Njeumi F, Nsangou C, Ndjend AG, Koga, Ostanello F, Pampiglione S (2002). Tunga penetrans in Cameroon Tunga penetrans au Cameroun. *Revue de Médicine Vétérinaire*.

[4] Winter B, Oliveira FA, Wilcke T, Heukelbach J, Feldmeier H (2009). Tungiasis-related knowledge and treatment practices in two endemic communities in northeast Brazil. *Journal of Infection in Developing Countries*.

[5] Collins G, McLeod T, Konfor NI, Lamnyam CB, Ngarka L, Njamnshi NL (2009). Tungiasis: a neglected health problem in rural cameroon. *International Journal of Collaborative Research on Internal Medicine and Public Health*.

[6] Veraldi S, Carrera C, Schianchi R (2000). Tungiasis has reached Europe. *Dermatology*.

[7] Leung AKC, Woo T, Robson WLM, Trotter MJ (2007). A tourist with tungiasis. *Canadian Medical Association Journal*.

[8] Veraldi S, Valsecchi M (2007). Imported tungiasis: a report of 19 cases and review of the literature. *International Journal of Dermatology*.

[9] Veraldi S, Schianchi R (1999). Guess what? Tungiasis. *European Journal of Dermatology*.

[10] Vennos E, Burke E, Johns C, Miller S (1995). Tungiasis. *Cutis*.

[11] Feldmeier H, Eisele M, Sabóia-Moura RC, Heukelbach J (2003). Severe tungiasis in underprivileged communities: case series from Brazil. *Emerging Infectious Diseases*.

[12] Heukelbach J, Sahebali S, Van Marck E, Sabóia Moura RC, Feldmeier H (2004). An unusual case of ectopic tungiasis with pseudoepitheliomatous hyperplasia. *The Brazilian Journal of Infectious Diseases*.

[13] Heukelbach J, Wilcke T, Eisele M, Feldmeier H (2002). Ectopic localization of tungiasis. *American Journal of Tropical Medicine and Hygiene*.

[14] Bauer J, Forschner A, Garbe C, Röcken M (2004). Dermoscopy of tungiasis. *Archives of Dermatology*.

[15] Marazza G, Campanelli A, Kaya G, Braun RP, Saurat JH, Piguet V (2009). Tunga penetrans: description of a new dermoscopic sign-the radial crown. *Archives of Dermatology*.

[16] Brothers WS, Heckmann RA (1980). Tungiasis in North America. *Cutis*.

[17] Heukelbach J, Eisele M, Jackson A, Feldmeier H (2003). Topical treatment of tungiasis: a randomized, controlled trial. *Annals of Tropical Medicine and Parasitology*.

[18] Heukelbach J, Franck S, Feldmeier H (2004). Therapy of tungiasis: a double-blinded randomized controlled trial with oral ivermectin. *Memorias do Instituto Oswaldo Cruz*.

[19] Cardoso A (1981). Generalized tungiasis treated with thiabendazole. *Archives of Dermatology*.

[20] Ugbomoiko US, Ariza L, Ofoezie IE, Heukelbach J (2007). Risk factors for tungiasis in Nigeria: identification of targets for effective intervention. *PLoS Neglected Tropical Diseases*.

